# Association between allergic conditions and colorectal cancer risk/mortality: a meta-analysis of prospective studies

**DOI:** 10.1038/s41598-017-04772-9

**Published:** 2017-07-17

**Authors:** Wangqian Ma, Jia Yang, Peiwei Li, Xinliang Lu, Jianting Cai

**Affiliations:** 1 Department of Gastroenterology, Second Affiliated Hospital, Zhejiang University College of Medicine, Hangzhou, 310009 China; 2 Department of Radiotherapy, Zhejiang Provincial People’s Hospital, Hangzhou, 310014 China

## Abstract

We aimed to assess the association between allergic conditions and risk/mortality of colorectal cancer (CRC). A systematic literature search was conducted using Pubmed and Embase to identify relevant studies. Prospective studies assessing the association between allergic conditions and risk/mortality of CRC were included. Risk ratios (RRs) were pooled with either a fixed- or a random-effects model according to heterogeneity. A total of 515379 participants and 10345 CRC cases from 12 studies were included in the analysis of CRC risk, while four studies with 1484741 individuals and 30040 CRC deaths were included in the analysis of CRC mortality. The pooled RR for the association between allergic conditions and CRC risk was 0.88 (95% CI 0.83–0.92). The inverse association was observed both in colon cancer (pooled RR = 0.83, 95% CI 0.72–0.97) and rectal cancer (pooled RR = 0.83, 95% CI 0.74–0.93). Moreover, no gender difference was observed in the analysis of CRC risk (for males, pooled RR = 0.88, 95% CI 0.81–0.96; for females, pooled RR = 0.88, 95% CI 0.82–0.95). And allergic conditions were also found to be inversely associated with CRC mortality (pooled RR = 0.88, 95% CI 0.83–0.92). In conclusion, the current meta-analysis provides further evidence that allergic conditions were inversely associated with CRC risk and mortality.

## Introduction

Colorectal cancer (CRC) is the third most common cancer in men and second most common cancer in women worldwide, with approximately 373,600 men and 320,300 women dying from CRC in 2012^1^. A number of lifestyle and environmental risk factors have been identified for CRC, including smoking, diet, obesity, diabetes, physical activity, and stain or aspirin use^2–4^. However, the exact mechanisms of CRC remain unclear. Allergic conditions including hay fever, asthma, atopic dermatitis, food allergy and drug allergy, may indicate an enhanced immune system, which may contribute to recognize and remove malignant cells and thus reduce risk of malignancies. Several studies reported a lower incidence of all malignancies among patients with allergic conditions compared with general population^5, 6^, while some other studies revealed a positive or non-significant association between allergic conditions and malignant disease^7, 8^. It was suggested that the association between allergic conditions and malignancy risk may be organ specific^9, 10^. For example, allergic conditions were reported to be associated with a lower risk of pancreatic cancer^9^ but a higher prostate cancer risk^10^.

In recent years, a number of studies investigated the association between allergic conditions and CRC risk or mortality with inconclusive results^11–14^. Considering these uncertainties, we performed a systematic review and meta-analysis to clarify the risk/mortality of CRC in patients with allergic conditions.

## Methods

This meta-analysis was planned, conducted and reported in adherence with the PRISMA statement^15^.

### Literature search and Study selection

A systematic literature search was performed in the Pubmed and Embase databases (up to January, 2015) without restrictions. The following search terms were used: (“colorectal cancer” or “colon cancer” or “rectal cancer” or “colorectal adenocarcinoma” or “colon adenocarcinoma” or “rectal adenocarcinoma”) AND (“allergies” OR “allergy” OR “allergic” OR “atopy” OR “atopic” OR “asthma” OR “allergic rhinitis” OR “hay fever” OR “atopic dermatitis” OR “atopic dermatitis” OR “hive” OR “eczema”). Moreover, reference lists of retrieved articles and relevant reviews were also scanned to avoid missing studies. We carefully examined the retrieved articles to exclude duplicate studies. Two authors independently screened the titles and abstracts of the articles selected from the initial search for potential inclusion, and full articles were reviewed subsequently to determine inclusion of eligible studies.

Criteria for inclusion were as follows: (1) the study was a prospective cohort or nested case-control study; (2) the study assessed the association between allergic conditions and risk and/or mortality of CRC; (3) relative risk (RR) or hazard ratio (HR) were reported or could be calculated. If data were reported in two or more studies, we only extracted the most detailed or recent information.

### Data extraction and quality assessment

We extracted the following information from each study: first author, year of publication, country, age and gender of participants, number of participants, number of CRC cases or deaths, variables adjusted for in the analysis, type of allergies evaluated in the study and RR (or HR) estimates with 95% CIs for CRC risk/mortality. RRs (HRs) reflecting the greatest degree of control for potential confounders were adopted in the meta-analysis. The Newcastle-Ottawa Scale was used to evaluate the quality of the included studies^16^. Studies were categorized into high quality if the score was 7 points or more, as medium quality if the score was 4 to 6 points and as low quality if the score was less than 4. The data extraction and quality assessment were conducted independently by two authors, and any discrepancies were resolved by discussion with a third investigator.

### Statistical analysis

The extent of heterogeneity across studies was assessed using the chi-square test and I^2^ test; *p* ≤ 0.05 and/or I^2^ > 50% were defined as significant heterogeneity^17^. Study-specific RR (HR) estimates for allergic conditions and CRC risk/mortality were pooled using a random-effects model if there was significant heterogeneity; otherwise, a fixed-effects model was applied. Subgroup analyses were further conducted for the association between allergic conditions and CRC risk, stratifying by gender, anatomical sites (colon or rectal cancer), geographic region and type of allergic conditions. Moreover, sensitivity and subgroup analyses were performed to explore the source of heterogeneity in the meta-analysis. To evaluate the publication bias risk, funnel plots and Egger’s linear regression method were conducted. Two-sided *p* values were calculated, with a *p* < 0.05 considered significant for all tests. All analyses were performed using the Stata software (version 11.0; StataCorp, College Station, TX, USA).

## Results

### Description of the included studies

**Figure 1:**
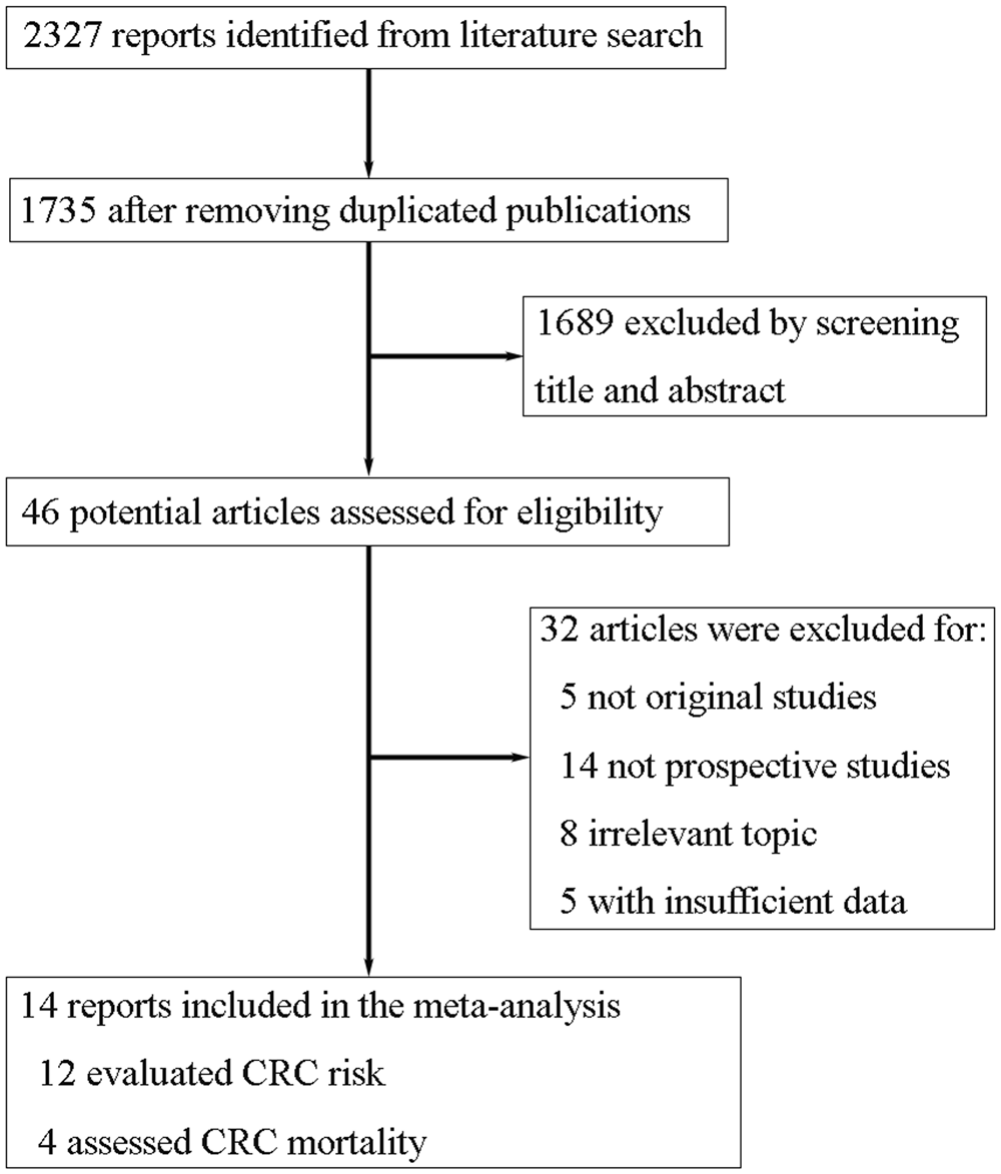
Flow diagram of studies through the retrieval and inclusion process in the meta-analysis. Two studies assessed both risk and mortality of CRC.

A literature search of Pubmed and Embase databases identified 2327 results, of which 46 were potentially eligible articles for further review. Among them, 32 papers were excluded because of the following reasons: the study was not an original study (n = 5), the study was not a prospective cohort or nested case-control study (n = 14), the study did not report the association between allergic conditions and CRC risk/mortality (n = 8), or an RR (HR) estimate was not reported and could not be calculated (n = 5). Finally, a total of 14 studies met the inclusion criteria and were included in this meta-analysis^6, 8, 11–14, 18–25^. Figure 1 shows the study selection process, whereas Table 1 indicates the characteristics of the included studies.

**Table 1:** Characteristics of the studies included in the meta-analysis.

Study	Design	Location	Age	Gender	No. of participants	Outcome of Interest	No. of CRC cases/deaths	Adjusted factors
Tambe/2015^11^	Cohort	USA	45–75	M/F	199112	Risk/Mortality	Cases:4834 Deaths:1363	Age, gender, smoking, educational level, BMI, NSAIDS use
Taghizadeh/2015^12^	Cohort	Netherlands	20–65	M/F	8465	Mortality	Deaths:134	Age, gender, FEV1 as % of predicted, BMI, place of residence
Skaaby/2014^13^	Cohort	Denmark	30–60	M/F	14894	Risk	Cases:211	Age, gender, education, physical activity, BMI, smoking, alcohol intake
Jacobs/2013^14^	Cohort	USA	Nr	M/F	Risk:174917 Mortality:2125283	Risk/Mortality	Cases:3365 Deaths:19202	Age, gender, race, education, BMI, physical activity, aspirin use, smoking
Chae/2012^18^	Cohort	USA	>=40	F	4600	Risk	Cases:37	Age, race, education, income, obesity, smoking, alcohol drinking, physical inactivity
Prizment/2011^19^	Cohort	USA	45–64	M/F	10675	Risk	Cases:242	Age, gender, race, education, BMI, smoking, alcohol, diabetes, WBC count, and fibrinogen
Prizment/2007^20^	Cohort	USA	55–69	F	22940	Risk	Cases:410	Age, smoking, total energy intake, calcium, red meat, multivitamin use, BMI, diabetes
Wang/2006^21^	Nested case-control	Germany	50–74	M/F	4748	Risk	Cases:477	Age, education, BMI, family history of cancer, smoking, alcohol
Gonzalez-Perez/2006^22^	Nested case-control	UK	20–79	M/F	25263	Risk	Cases:436	Age, gender, calendar year, BMI, alcohol, smoking, prior comorbidities, health services utilization, use of aspirin, NSAID, and paracetamol
Eriksson/2005^8^	Cohort	Sweden	Median:32	M/F	13811	Risk	Cases:25	Age, sex
Turner/2005^6^	Cohort	USA	>30	M/F	1102247	Mortality	Deaths:9341	Age, gender, race, smoking, education, BMI, exercise, alcohol, aspirin use, intakes of vegetables, red meat, and fiber, multivitamin use, family history
Talbot-Smith/2003^23^	Cohort	Australia	Nr	M/F	3308	Risk	Cases:67	Age, gender, smoking, BMI
Mills/1992^24^	Cohort	USA	Nr	M/F	34198	Risk	Cases:196	Age, gender, smoking, time since last physician contact
McWhorter/1988^25^	Cohort	USA	25–74	M/F	6913	Risk	Cases:45	Age, gender, race, smoking

Nr: not reported; BMI: body mass index; NSAIDS: non-steroidal anti-inflammatory drugs.

Twelve of the 14 included studies evaluated CRC risk in patients with allergic conditions, while 4 investigated CRC mortality (several studies reported both CRC risk and mortality). All the studies were conducted in Western countries: eight in USA, five in European countries and one in Australia. Among the included studies, twelve were cohort studies and the other two were nested case-control studies. Two studies enrolled female participants only, whereas the other studies included both males and females. Sample size of the included studies ranged from 3308 to 199112. All the included studies investigated both colon and rectal cancer. The methodological quality of the included studies was shown in Supplementary Table 1.

### Association between allergic conditions and risk of CRC

**Figure 2:**
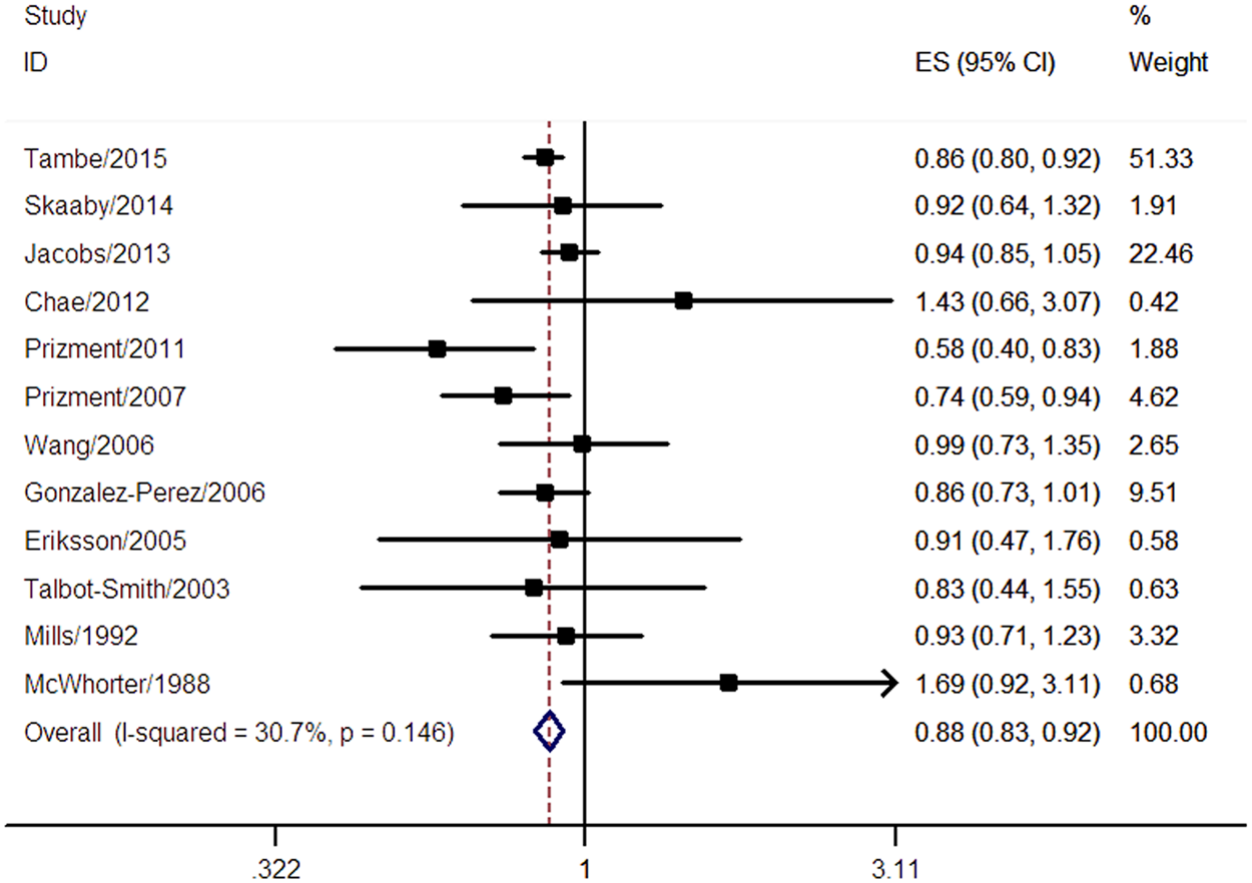
Meta-analysis of the association between allergic conditions and risk of CRC. A fixed-effect model was used in the analysis and a significant inverse association between allergic conditions and risk of CRC was observed.

A total of 12 studies assessed the association between allergic conditions and risk of CRC. These studies comprised 515379 participants, of whom 10345 developed CRC. Suggested by the pooled result, individuals with allergic conditions were at a lower risk of CRC (pooled RR = 0.88, 95% CI 0.83–0.92) (Fig. 2). We observed no significant heterogeneity across the included studies (I^2^ = 30.7%, *p* = 0.146) (Fig. 2). The Begg’s funnel plot (*p* = 0.537) (Supplementary Figure 1) and Egger’s test (*p* = 0.591) indicated no evidence of publication bias.

Subgroup analyses revealed that individuals with allergic conditions had a lower risk of both colon cancer (pooled RR = 0.83, 95% CI 0.72–0.97) and rectal cancer (pooled RR = 0.83, 95% CI 0.74–0.93). We also found an inverse association between allergic conditions and CRC risk both in males (pooled RR = 0.88, 95% CI 0.81–0.96) and in females (pooled RR = 0.88, 95% CI 0.82–0.95). With regard to specific allergic conditions, skin allergy (pooled RR = 0.76, 95% CI 0.56–1.02), hay fever (pooled RR = 0.94, 95% CI 0.85–1.04), and asthma (pooled RR = 0.92, 95% CI 0.77–1.11) were not significantly associated with CRC risk. In the subgroup analyses, significant heterogeneity was observed in the analysis of colon cancer (I^2^ = 51.0%, *p* = 0.069) and in the studies conducted in the USA (I^2^ = 58.9%, *p* = 0.024). Sensitivity analyses were performed to explore the potential source of heterogeneity. We found that after excluding the study by Jacobs *et al*.^14^, no significant heterogeneity was found in the analysis of colon cancer (I^2^ = 44.9%, *p* = 0.123), while heterogeneity in the analysis of studies from USA decreased after excluding the study by Prizment *et al*.^19^ (I^2^ = 44.3%, *p* = 0.110). The results of subgroup analyses were shown in Table 2.

**Table 2:** Subgroup analysis of the association between allergic conditions and colorectal cancer risk.

Factor	No. of Studies	Pooled RR (95% CI)	Heterogeneity	
			I^2^ (%)	*p*
Cancer type				
Colon cancer	6	0.83 (0.72–0.97)	51.0	0.069
Rectal cancer	6	0.83 (0.74–0.93)	0	0.538
Gender				
Male	3	0.88 (0.81–0.96)	0	0.616
Female	5	0.88 (0.82–0.95)	31.3	0.213
Geographic Region				
USA	7	0.88 (0.75–1.02)	58.9	0.024
Other Countries	5	0.89 (0.78–1.01)	0	0.949
Allergic condition				
Skin allergy	3	0.76 (0.56–1.02)	0	0.682
Hay fever	5	0.94 (0.85–1.04)	0	0.947
Asthma	5	0.92 (0.77–1.11)	0	0.435

### Association between allergic conditions and CRC mortality

**Figure 3:**
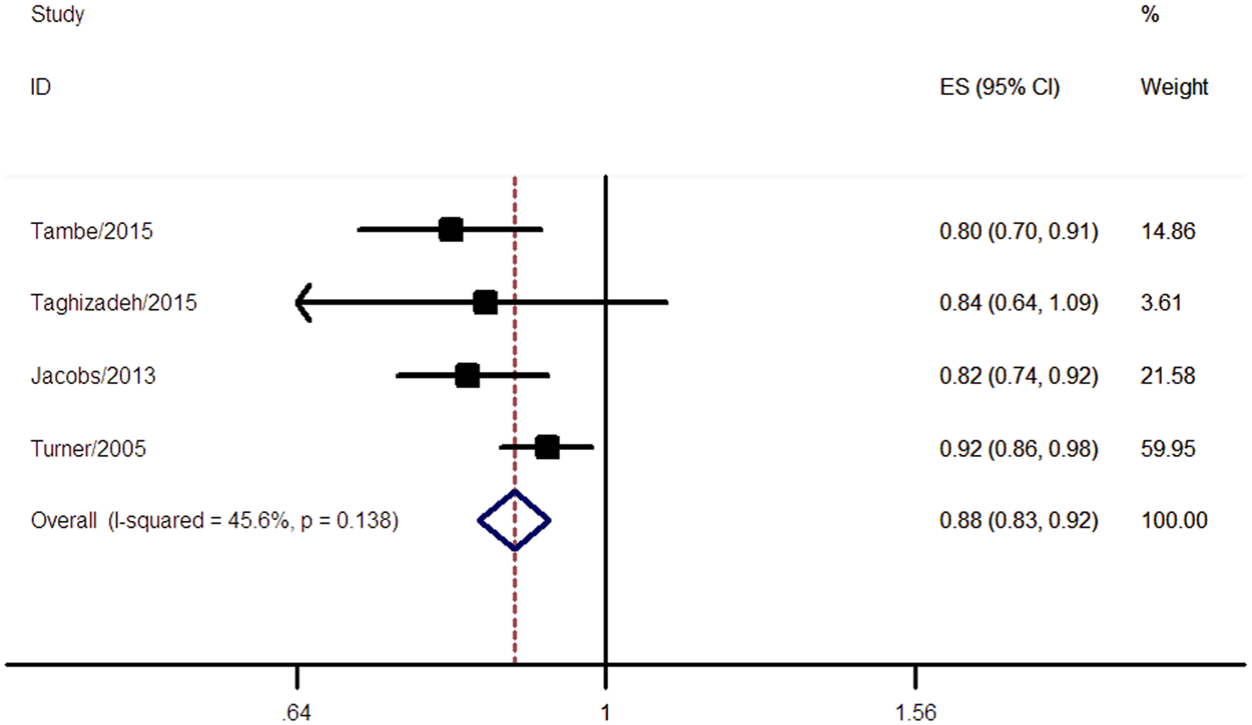
Meta-analysis of the association between allergic conditions and mortality of CRC. A fixed-effect model was used in the analysis and a significant inverse association between allergic conditions and mortality of CRC was observed.

Four studies with 1484741 participants and 30040 CRC deaths were included in the analysis of CRC mortality. Allergic conditions were associated with a 12% decrease in CRC mortality (pooled RR = 0.88, 95% CI 0.83–0.92) without significant heterogeneity (I^2^ = 45.6%, *p* = 0.138) (Fig. 3). The Begg’s funnel plot (*p* = 0.734) (Supplementary Figure 2) and Egger’s test (p = 0.295) found no evidence of publication bias. However, because of the limited number of included studies, the possibility of publication bias could not be fully ruled out.

## Discussion

Although the association between allergic conditions and CRC risk/mortality has long been investigated, no clear conclusion has been made so far. Our meta-analysis systematically assessed this association, including 14 prospective cohort or nested case-control studies reporting risk and/or mortality of CRC. Compared with the general population, individuals with allergic conditions were at a lower risk of CRC risk and mortality. Suggested by the subgroup analyses, an inverse association with allergic conditions were observed both in colon cancer and in rectal cancer. Moreover, our analysis also suggested that both males and females with allergic conditions had a lower risk of CRC. We found that skin allergy, hay fever and asthma were not significantly associated with CRC risk, whereas other allergic conditions such as atopic dermatitis were not analyzed because of limited data.

A number of researches have explored the effect of allergic conditions on malignant diseases, however, no clear conclusion has emerged. The association between allergic conditions and malignant diseases appeared to be different according to cancer type. As indicated by a meta-analysis, any allergy was associated with 21% decrease of pancreatic cancer risk (pooled OR = 0.79, 95% CI 0.62–1.00)^9^. Allergic conditions were also inversely associated with risk of head and neck cancer^26^. However, patients with allergic conditions were at a higher risk of lymphoma and prostate cancer, compared with the general population^10, 27^. Besides, specific allergic conditions may have different effect on cancer risk. For example, hay fever and allergy to animals, but not other allergies or asthma, were associated with decreased pancreatic cancer risk^9^.

Though a large number of studies have investigated the role of allergic conditions in cancer, the mechanisms remain unclear^10, 13, 28, 29^. Allergic conditions appear to have dual roles in CRC^28, 30^. Allergies may cause chronic inflammation and stimulate cell growth, which can predispose an individual to malignant disease^28, 30^. On the other hand, two hypotheses, “immunosurveillance” hypothesis and “prophylaxis” hypothesis, have been proposed to explain the inverse association between allergic conditions and cancer^28^. The “immunosurveillance” hypothesis proposes that people with allergic conditions have heightened immune system, which contributes to detect and eradicate pre-malignant cells and thus reduces cancer risk^31, 32^. Another potential explanation, known as prophylaxis hypothesis, asserts that allergic responses in mucosal surfaces can lead to more rapid clearance of toxins, pathogens, and foreign particles before they initiate carcinogenesis^28^. The protective effect of allergic conditions on CRC could also be explained by the antitumor effect of type I immunoglobulin E-mediated immune activity^11^. Atopic allergens can be taken into the digestive system and lead to type I immunoglobulin E-mediated hypersensitivity reactions^33^. A previous study found that coculture of eosinophils with colorectal carcinoma cells resulted in secretion of eosinophil cationic protein and granzyme A, which exerts eosinophil tumoricidal activity toward CRC cells^34^. Besides, IgE can bind to cell surface IgE receptors such as CD23 and FceRI, which engage several types of effector cells against cancer^35^. Moreover, it is also indicated that treatment with tumor-specific mouse IgE antibody could inhibit human colorectal tumor xenograft growth^36^. More studies are warranted to further clarify the exact mechanisms for the antitumor effect of allergic conditions.

A previous meta-analysis evaluated the association between atopic diseases and breast, prostate, and colorectal cancers, finding no significant association between atopic diseases and CRC risk^37^. However, most of the included studies were case-control studies, while only 8 were cohort/nested case-control studies (6 were prospective and 2 were retrospective). Retrospective studies tend to have biases in control selection and recall of former exposure to risk factors^38^. Prospective studies can avoid these problems, thus evidence from prospective studies is generally considered to be stronger than that from retrospective studies. In the current meta-analysis, all the included studies were prospective cohort or nested case-control studies with a substantial number of participants, which increased the statistical power.

However, there are still several limitations that should be considered when interpreting the results. First, though most of the studies included in the current meta-analysis were adjusted for other known risk factors of CRC (shown in Table 1), the confounders in the included studies were different and there is a possibility that control of confounders was inadequate. For instance, compared with those without allergic conditions, individuals with allergic conditions may have higher endoscopy rates^11^. Endoscopy use could reduce CRC risk by removal of adenomas, while it may also increase the rate of CRC detection. This may lead to over-estimation or under-estimation in the effect of allergic conditions. Second, the types of allergic conditions and the diagnostic methods were different across the included studies. An inverse association between any allergic condition and CRC risk/mortality was observed, while no significant association was indicated for specific allergic conditions including asthma, hay fever and skin allergy. Other kinds of allergies, such as food allergy, were not evaluated because of the limited data. An individual participant data (IPD) meta-analysis might be helpful to clarify this issue. Finally, all the included studies were conducted in Western countries, thus, the results of the current study should be interpreted with caution for population from other countries.

In conclusion, the current meta-analysis of prospective studies suggested an inverse association between allergic conditions and risk/mortality of CRC. The protective effect of allergic conditions was observed both in colon cancer and rectal cancer. These findings further highlight a potential protective role of the reactive immune system in CRC.

## Electronic supplementary material

Supplementary File
