# Supplementary material for: Association between allergic conditions and colorectal cancer risk/mortality: a meta-analysis of prospective studies
Source: Sci Rep. 2017 Jul 17;7:5589. doi: 10.1038/s41598-017-04772-9 (PMC5514030; doi:10.1038/s41598-017-04772-9)

Association between allergic conditions and colorectal cancer risk/mortality: a meta-analysis of prospective studies

Wangqian Ma[1], Jia Yang[2], Peiwei Li[1], Xinliang Lu[1]*, Jianting Cai[1]*

[1] Department of Gastroenterology, Second Affiliated Hospital, Zhejiang University College of Medicine, Hangzhou, 310009 China [2] Department of Radiotherapy, Zhejiang Provincial People's Hospital, Hangzhou, 310014 China

(Running Title: Allergic conditions and colorectal cancer risk/mortality)

*: Corresponding Authors:

Xinliang Lu, MD, Department of Gastroenterology, Second Affiliated Hospital, Zhejiang University College of Medicine, Hangzhou, 310009 China; Tel/Fax: 86-0571-87022776; E-mail: xinliang.lu@yahoo.com

Jianting Cai, MD, Department of Gastroenterology, Second Affiliated Hospital, Zhejiang University College of Medicine, Hangzhou, 310009 China; Tel/Fax: 86-0571-87022776; E-mail: jtcai6757@163.com

We declare that we have no conflict of interest.

Supplementary Table 1 Quality assessment of the included studies

Study	Q1	Q2	Q3	Q4	Q5	Q6	Q7	Q8	Score
Tambe/2015[11]	b	a	c	a	ab	b	a	b	8
Taghizadeh/2015[12]	a	a	a	a	ab	d	a	d	7
Skaaby/2014[13]	b	a	a	a	ab	b	a	c	8
Jacobs/2013[14]	a	a	c	a	ab	b	a	b	8
Chae/2012[18]	b	a	c	a	ab	c	a	d	6
Prizment/2011[19]	b	a	a	a	ab	b	a	d	8
Prizment/2007[20]	c	a	c	a	ab	b	a	b	7
Wang/2006[21]	a	b	b	a	ab	a	a	b	6
Gonzalez-Perez/2006[22]	c	b	a	a	ab	d	a	b	5
Eriksson/2005[8]	c	b	a	a	ab	b	a	d	6
Turner/2005[6]	b	a	c	a	ab	d	a	b	7
Talbot-Smith/2003[23]	b	a	c	a	ab	b	a	d	7
Mills/1992[24]	c	a	c	a	ab	b	a	d	6
McWhorter/1988[25]	b	a	c	a	ab	a	a	b	8

Questions for cohort study (one star is defined as one score)

Q1: Representativeness of the exposed cohort

a) truly representative of the average population in the community* b) somewhat representative of the average population in the community*

c) selected group of users d) no description of the derivation of the cohort

Q2: Selection of the non exposed cohort

a) drawn from the same community as the exposed cohort* b) drawn from a different source

c) no description of the derivation of the non-exposed cohort

Q3: Ascertainment of exposure

a) secure record* b) structured interview*

c) written self-report d) no description

Q4: Demonstration that outcome of interest was not present at start of study

a) yes* b) no

Q5: Comparability of cohorts on the basis of the design or analysis

a) study controls for age* b) study controls for any additional factor Outcome*

Q6: Assessment of outcome

a) independent blind assessment* b) record linkage*

c) self-report d) no description

Q7: Was follow-up long enough for outcomes to occur

a) yes* b) no

Q8: Adequacy of follow up of cohorts

a) complete follow up - all subjects accounted for *

b) subjects lost to follow up unlikely to introduce bias - small number lost > 70 % follow up, or description provided of those lost*

c) follow up rate < 70% and no description of those lost

d) no statement

Questions for case-control study (one star is defined as one score)

Q1: Is the case definition adequate?

a) yes, with independent validation* b) yes, eg record linkage or based on self-reports c) no description

Q2: Representativeness of the cases

a) consecutive or obviously representative series of cases* b) potential for selection biases or not stated

Q3: Selection of Controls

a) community controls* b) hospital controls c) no description

Q4: Definition of Controls

a) no history of disease (endpoint) * b) no description of source

Q5: Comparability of cases and controls on the basis of the design or analysis
a) study controls for age* b) study controls for any additional factor*
Q6: Ascertainment of exposure
a) secure record* b) structured interview where blind to case/control status
c) interview not blinded to case/control status d) written self-report or medical record only
e) no description
Q7: Same method of ascertainment for cases and controls
a) yes* b) no
Q8: Non-Response rate
a) same rate for both groups* b) non respondents described c) rate different and no designation

Supplementary Figure 1 Funnel plots for the analysis of CRC risk

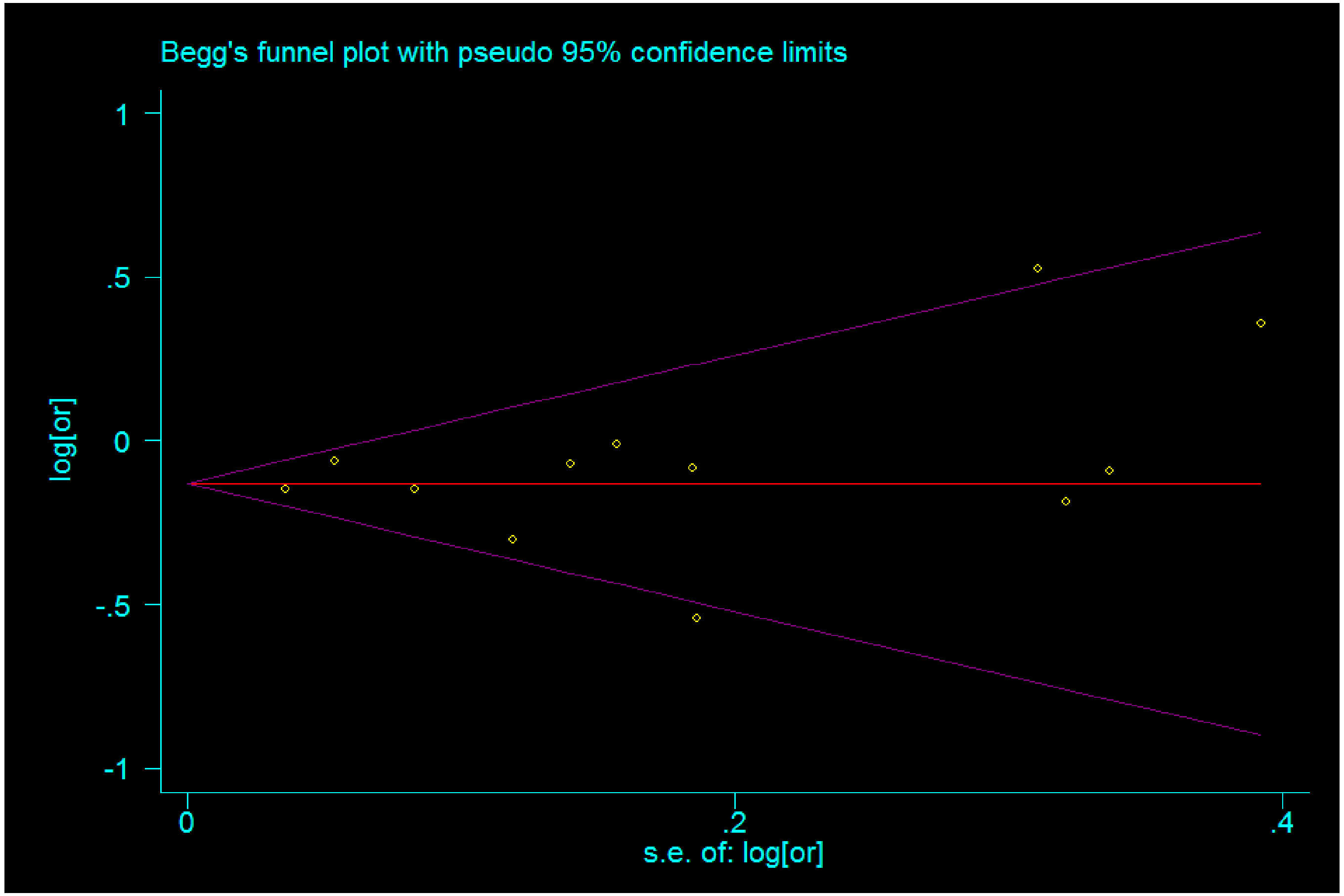

Supplementary Figure 2 Funnel plots for the analysis of CRC mortality

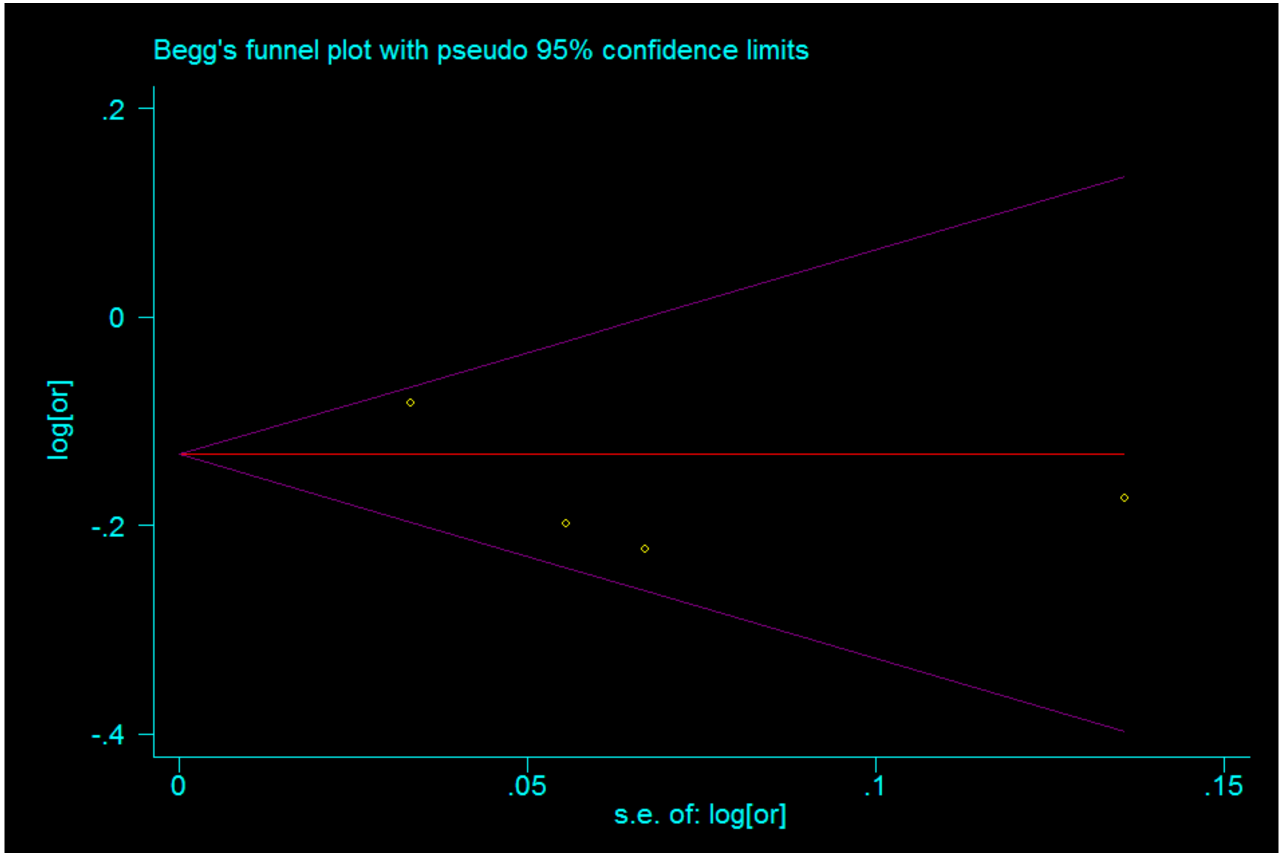

Supplement: Supplementary file 1 — Supplementary File [file 41598_2017_4772_MOESM1_ESM.pdf]
